# ADAMTS7 Attenuates House Dust Mite-Induced Airway Inflammation and Th2 Immune Responses

**DOI:** 10.1007/s00408-022-00538-x

**Published:** 2022-05-03

**Authors:** Anil Kumar Jaiswal, Amarjit Mishra

**Affiliations:** Laboratory of Lung Inflammation, Department of Pathobiology, College of Veterinary Medicine, Auburn University, 254 Greene Hall, 1130 Wire Road, Auburn, AL 36849 USA

**Keywords:** ADAMTS7, Lung dendritic cells, Allergic airway inflammation (AAI), Type 2 immune response

## Abstract

**Purpose:**

ADAMTS7 is a secreted metalloproteinase enzyme and proteoglycan associated with the early progression of coronary artery disease. However, there is limited information regarding the role of ADAMTS7 in lung adaptive immunity and inflammation. Thus, we sought to assess whether ADAMTS7 expression in the lung modulates house dust mite (HDM)-induced airway inflammation and Th2 immune response.

**Methods:**

The role of ADAMTS7 in HDM-induced airway disease was assessed in ADAMTS7-deficient (ADAMTS7^−/−^) mice and compared with the wild-type control mice by flow cytometry, ELISA, and histopathology. Furthermore, the antigen priming capability of dendritic cells (DC) was determined ex vivo by employing coculture with CD4^+^ OT-II cells.

**Results:**

ADAMTS7^−/−^ mice develop an augmented eosinophilic airway inflammation, mucous cell metaplasia, and increased Th2 immune response to inhaled HDM. In addition, allergen uptake by lung DC and migration to draining mediastinal lymph node were significantly increased in ADAMTS7^−/−^ mice, which shows an enhanced capacity to mount allergen-specific T-cell proliferation and effector Th2 cytokine productions. We propose that the mechanism by which ADAMTS7 negatively regulates DC function involves attenuated antigen uptake and presentation capabilities, which reduces allergic sensitization and Th2 immune responses in the lung.

**Conclusion:**

In aggregate, we provide compelling evidence that ADAMTS7 plays a pivotal role in allergic airway disease and Th2 immunity and would be an attractive target for asthma.

## Introduction

Allergic airway inflammation associated with asthma affects ~ 7% of the United States population, with high morbidity and cost burden to the health care system [1, 2]. The heterogeneity of asthma based on the clinical manifestation of Th2-high/eosinophilic and Th2-low/neutrophilic airway inflammation stems from underlying complex molecular mechanisms that complicate therapy response and impact health outcomes [3–6]. Dendritic cells (DC) are generated from a rare heterogeneous population of hematopoietic cells that co-evolved with the formation of the adaptive immune system. Lung DC tailor allergic sensitization and Th2 adaptive immune responses to match inhaled aeroallergens, such as house dust mite (HDM), by constantly sampling the allergen uptake and migrating to a nearby T-cell-rich zone draining lymph nodes (DLn) to present the lung-derived antigens. DC play pivotal roles in initiating Th2 cell differentiation and effector cytokines productions to mount airway inflammation and allergic responses [7, 8]. The salient features of experimental asthma include increased pulmonary eosinophilia and lymphocytosis, increased mucus production by goblet cells, and structural remodeling of the airway wall. However, in this context, limited or conflicting data exist regarding the function of secreted matrix metalloproteinases in allergen-mediated immune responses driving airway inflammation in asthma.

A disintegrin and metalloproteinase with thrombospondin type I motif 7 (ADAMTS7) is a secreted metalloproteinase enzyme with proteolytic activity and has been associated with diseases including early progression of atherosclerosis, rheumatoid arthritis, and inflammation of intravertebral discs [9–12]. The proteolytic activity of ADAMTS7 is essential for the regulated cleavage of extracellular matrix (ECM) proteins, VSMC migration, and atherogenic effects, thereby driving disease progression [13–15]. Moreover, ADAMTS7 and its close paralog ADAMTS12 cooperatively modulate tendon-specific collagen fibrillogenesis and protect from heterotropic ossification in mice [10]. ADAMTS7 transgenic knockout mice are developmentally normal and viable; however, develop less atherosclerotic plaques in the hyperlipidemic mouse model [15]. Recent Longevity Genomics studies have reported the association of ADAMTS7-related genetic variants with predicted lung function (FEV1) in the elderly [16]. Importantly, allelic variation of ADAMTS7 at rs7178051 renders strong cardioprotection in non-smokers compared to smokers [17, 18]. However, the potential role of ADAMTS7 in allergic airway inflammation and Th2 immunity is yet to be defined.

This study investigated whether ADAMTS7 contributes to DC-mediated allergic sensitization and airway Th2 inflammation. Using murine models of allergic asthma, we demonstrate that ADAMTS7 is induced in response to HDM sensitization resulting in Th2 eosinophilic airway inflammation. First, we created an ADAMTS7-deficient mouse to characterize the role of ADAMTS7 expression in HDM-induced airways disease. We demonstrate that HDM-challenged ADAMTS7^−/−^ mice displayed a phenotype of augmented allergic sensitization and enhanced Th2 immune responses to HDM that were associated with eosinophilic airway inflammation. HDM restimulation of draining DLn cells showed increased IL5 and IL13 productions, which are critical regulators of eosinophilic airway inflammation. In addition, we show that DC obtained from ADAMTS7^−/−^ mice have an elevated allergen uptake/migration and antigen-presenting capability to CD4^+^ T-cells, thereby accelerating Th2 effector functions.

## Methods

### Mice

ADAMTS7^−/−^ (Adamts7 ^tm1a (KOMP) Wtsi^ knockout first/conditional ready) (Stock number 046487-UCD) [19] mouse was obtained from UC Davis and was bred in-house. C57BL/6NJ wild-type (WT) control mice, B6.Cg-Tg (Tcra Tcrb) 425Cbn/J (transgenic mice expressing MHCII-restricted TCR that binds to OVA_323-339_ peptide antigen) [20] were obtained from the Jackson Laboratories (Bar Harbor, ME). The mutant ADAMTS7^−/−^ mice are viable and fertile. All animals were housed in individually ventilated cages under specific pathogen-free conditions at the animal facility, Auburn University, and used for experiments between 6 and 12 weeks unless otherwise stated with age-matched groups in every individual experiment. Experimental protocols of the murine model of allergy and asthma were approved by the Animal Care and Use Committee of the Auburn University, Auburn, AL.

### Murine Models of Airway Inflammation

**Fig. 1:**
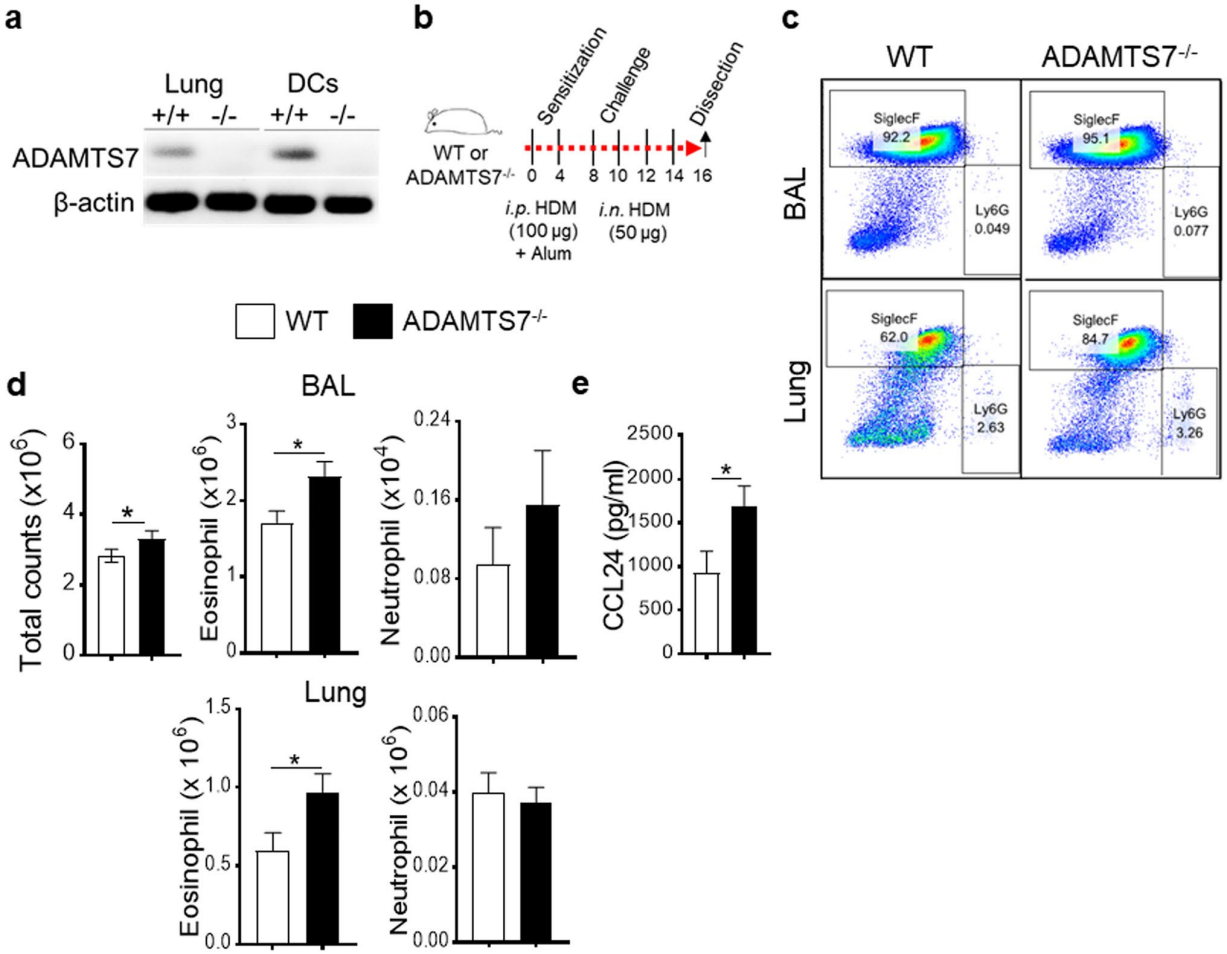
ADAMTS7-deficient mice develop augmented HDM-induced airway inflammation. **a** Representative PCR analysis of genomic DNA isolated from WT and ADAMTS7^−/−^ lungs and DC. The absence of PCR product indicates ADAMTS7 deletion in ADAMTS7^−/−^ mice. **b** Diagram shows the HDM administration (*i.p.,* intraperitoneal; *i.n.* intranasal) and analysis schedule. **c** Dot plots of BAL and lung cells (from a representative individual of indicated groups of mice and **d** quantitation of flow cytometry analysis showing the number of total cells (CD45 +), eosinophils (CD11b^+^ Siglec F^+^), and neutrophils (CD11b^+^ Ly-6G^+^) in BAL (top) and lung (bottom). **e** BAL levels of C–C chemokine CCL24 from WT and ADAMTS7^−/−^ mice. Results show the mean ± S.E. and represent two or more independent experiments (*n* = 8–10 mice, unpaired *t-*test, *P < 0.01)

For the chronic HDM model, animals received 100 μg low-endotoxin HDM (*Dermatophagoides pteronyssinus* extract, Greer Laboratories, Lenoir, NC) with alum intraperitoneally (*i.p.*) for sensitization on day0 and day4. Mice were HDM-challenged (50 µg per mouse in a volume of 40 μl sterile PBS) on days 8, 10, 12, and 14 before harvest on day 16, as indicated in the scheme in Fig. 1b.

### BAL and Lung Histopathology

Bronchoalveolar lavage (BAL) was collected with 0.5 ml PBS, and red blood cells were lysed with ACK buffer for 2 min at 4 °C. BAL cells were re-suspended in 0.3 ml RPMI-1640 containing 10% FBS, and cell counts were performed using a hemocytometer, while differential cell counts were performed by flow cytometry. Lungs were fixed in 10% formalin for 24 h, dehydrated through gradient ethanol, and embedded in paraffin. Sections were cut sagittally at a thickness of 5 µm and stained with hematoxylin, eosin, and periodic acid Schiff (PAS). Semi quantification of mucous cell metaplasia was performed by enumerating the number of PAS-positive airways (large-conducting, medium-central, and small-distal) within a representative lung section reported as the percentage of airways that had PAS-positive cells, as previously described [21].

### Flow Cytometry

Multiparameter analysis was performed on an LSRII (BD Biosciences, USA) equipped with 407, 488, and 633 LASER lines using DIVA 8 software and analyzed with the Flow Jo software version 10.7.1 (Treestar, San Carlos, CA). Using FSC/SSC plot, cellular debris was excluded. Eosinophils (CD45^+^ CD11b^+^ SiglecF^+^ Ly6G^−^) and neutrophils (CD45^+^ CD11b^+^ SiglecF^−^Ly6G^+^) in BAL and lung were identified using the following antibodies: rat anti-mouse CD45 efluor 450 (clone 30-F11), CD11b-APCCy7 (clone M1/70) Ly6G-APC (clone 1A8), and SiglecF-PE-Texas Red (clone 1RNM44N). For analysis of intracellular cytokines, cells were first stained with surface antigens against rat anti-mouse CD45 efluor 450 (clone 30-F11), CD3-Alexa Fluor 647 (clone 17-A2), and CD4-APC-eFluor 780 (clone GK1.5) and fixed with IC Fixation Buffer (eBiosciences) for 30 min, followed by a wash with Perm/Wash buffer (BD). Cells were then re-suspended and reacted to monoclonal antibody cocktails of rat anti-mouse IL-13 Alexa Fluor 488 (eBio13A) and IL-17 eFluor 450 (eBio17B7) in Perm/Wash buffer (50 μl) for 30 min at room temperature. Viable CD3^+^/CD4^+^ cytokine^+^ cells were quantified using FMO (fluorescence minus one) as controls.

### Ex Vivo Cultures and Restimulation of DLn Cells

Single-cell suspensions from mediastinal lymph nodes (DLn) of WT and ADAMTS7^−/−^ mice were prepared using a sterile RPMI medium containing 10% FBS. DLn cells were counted and cultured at a density of 0.3 × 10^6^ cells ml^−1^ in round-bottom 96-well plates and pulsed with HDM (100 µg ml^−1^) for 96 h at 37 °C in RPMI medium containing 10% FBS. IL5 and IL13 in the supernatants were determined using sandwich ELISA kits (ThermoFisher Scientific) with sensitivity limits of 4 pg ml^−1^ for IL-5 and IL13.

### Antigen Uptake/Migration and Presentation Assay

WT and ADAMTS7^−/−^ mice received intranasal administration of Alexa fluorophore 647-labeled OVA (100 µg per sensitization) dissolved in 40 µl of PBS. At 72 h after sensitization, migrating DC were enumerated in digested lungs and DLn as SiglecF^−^ CD11c^+^ MHCII^hi^ AF647-OVA^+^ cells by flow cytometry [22]. Ex vivo antigen-specific T-cell proliferation was assessed using CFSE-labeled splenic CD4^+^ T-cells obtained from naïve [B6.Cg-Tg(Tcra Tcrb)425Cbn/J] transgenic mice (Jackson Laboratories, Bar Harbor, ME) in coculture to determine the antigen presentation capability of DCs. The mice express a transgenic MHCII-restricted TCR and recognize the OVA peptide antigen [8]. Splenic naive CD4^+^ T-cells were purified using EasySep Mouse CD4^+^ T-Cell Isolation Kit (Stem cells, Vancouver, CA) and were labeled with 5 µM CFDA-SE (carboxyfluorescein diacetate succinimidyl ester; Cayman Chemical, MI, USA) in DPBS for 20 min at 37 °C. Bone marrow-derived dendritic cells (BMDC) from WT and ADAMTS7^−/−^ mice were pulsed overnight with 5 μg ml^−1^ of OVA_323-339_ peptide (AnaSpec, Fremont, CA, USA) or PBS. 1 × 10^5^ OVA peptide-specific CD4^+^ OT-II cells were cocultured with 2 × 10^4^ CD11c^+^ BMDC for 4 days in 96-well round-bottom plates. T-cell proliferation was quantified by flow cytometry using the CFSE dye dilution method. The FlowJo Proliferation Platform analyzed gated CD4+T-cells as percent divided. IL4 and IL13 released into the culture medium were measured using ELISA. Additionally, cells were stained with CD3-Alexa Fluor 647 (clone 17-A2) and CD4-APC-eFluor 780 (clone GK1.5) before incubation with GATA3 eFluor 450 (clone TWAJ) for flow cytometry.

### Statistics

Data were analyzed using Graph Pad Prism version 7.0b and are presented as mean ± SEM. A one-way ANOVA with Tukey’s multiple comparison test and a Mann–Whitney U test for nonparametric comparison or student *t*-test for comparison of two groups were used for the analyses. A *P* value < 0.05 was considered significant.

## Results

### ADAMTS7 Regulates the Development of HDM-Induced Chronic Allergic Airway Inflammation

**Fig. 2:**
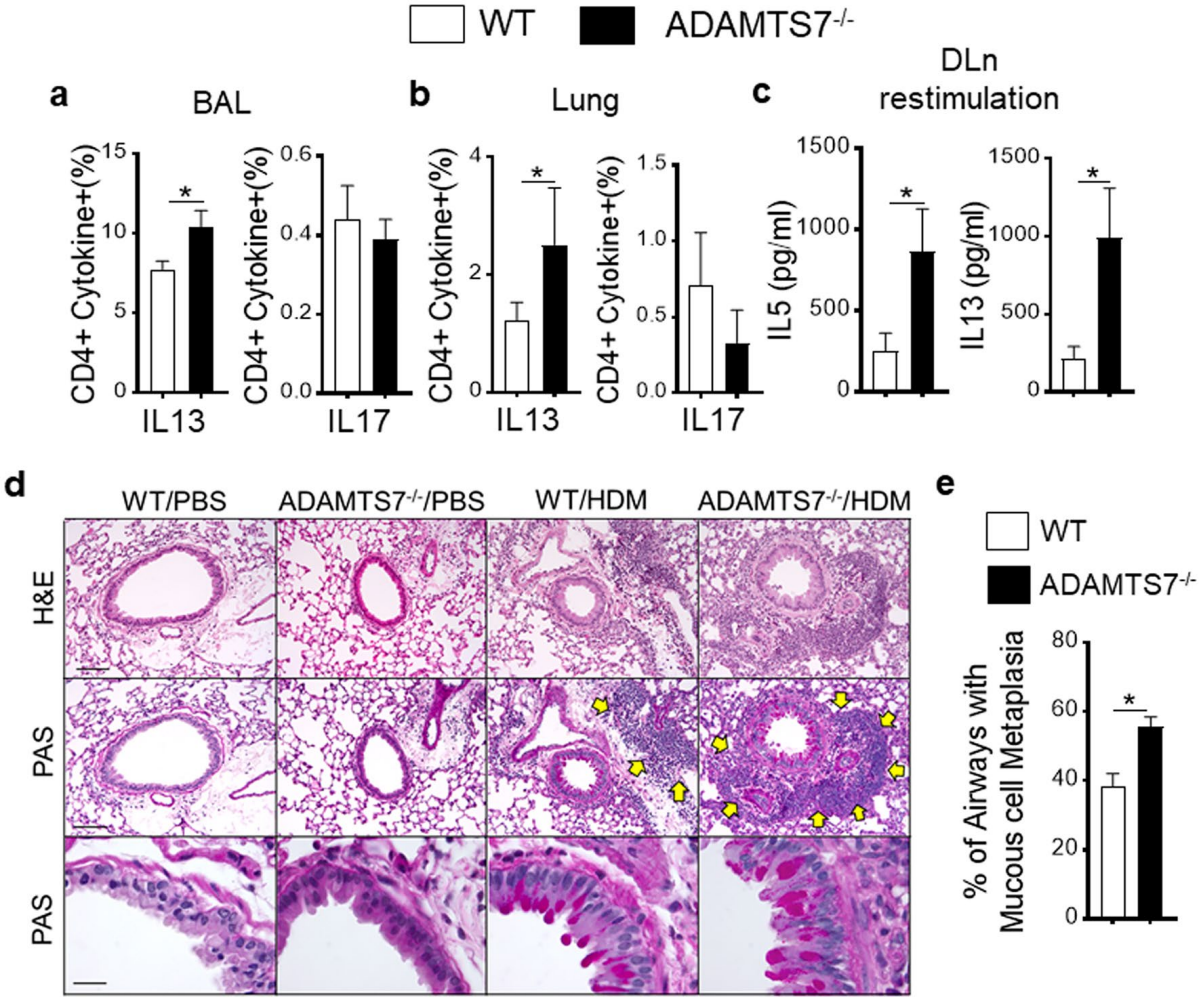
ADAMTS7^−/−^ develops increased Th2 immune response to inhaled HDM. Frequency of CD4+IL13+and CD4+IL17+T-cells in **a** BAL and **b** lung; and **c** cytokine secretion by ex vivo cultures of DLn cells that had been re-stimulated with HDM (100 µg/ml). Results show the mean ± S.E. and represent two or more independent experiments (*n* = 8–10 mice, unpaired *t-*test, *P < 0.01). **d** Images show histopathological sections of HDM-challenged lung from WT or ADAMTS7^−/−^ stained with H& E and PAS. *Scale bars*, 100 μm for × 100 and 50 μm for × 400 images. **e** Frequency of PAS+airways from HDM-challenged WT or ADAMTS7^−/−^mice. Results show the mean ± S.E. and represent two or more independent experiments (*n* = 6 mice, unpaired *t-*test, *P < 0.01)

ADAMTS7 knockout mice strain was obtained from Mutant Mouse Resource and Research Center (MMRRC) [19] and bred in-house. The expression of ADAMTS7 in the lung and isolated DC was confirmed by PCR of genomic DNA (Fig. 1a). Next, experiments were conducted with multiple nasal HDM challenges to wild-type (WT) and ADAMTS7^−/−^ mice (Fig. 1b). Lung immune responses and airway inflammation were evaluated employing flow cytometry (Fig. 1c) 24 h after the last HDM challenge. The results demonstrate a significant increase in total BAL cells and lung inflammatory cells in HDM-challenged ADAMTS7^−/−^ mice, which reflected a selective increase in eosinophils (Fig. 1d). However, the numbers of neutrophils were not altered between WT and ADAMTS7 knockout mice. The C–C chemokine CCL24, which recruits eosinophils, was significantly increased in BAL from HDM-challenged ADAMTS7^−/−^ mice than in control WT (Fig. 1e). Chronic allergen stimulation with HDM showed significant infiltrations of CD4^+^ effector T-cells expressing high IL13 cytokine in ADAMTS7^−/−^ mice compared to WT mice in BAL and the lung (Fig. 2a, b). However, there were no differences in IL17 expression between the two genotypes. Restimulation of DLn cells with HDM isolated from inflamed ADAMTS7^−/−^ mice showed a significant increase in IL5 and IL13 Th2 cytokines production in the culture supernatant compared with the HDM-challenged WT mice (Fig. 2c). Moreover, we found increased peribronchial inflammatory cell infiltrates, and PAS+airways in HDM-challenged ADAMTS7^−/−^ mice lungs compared with WT mice controls (Fig. 2d, arrow indicates peribronchial cell infiltrations). Overall, these results showed that ADAMTS7^−/−^ mice have a phenotype of increased eosinophilic airway inflammation, mucous cell metaplasia, and Th2-mediated immune responses in HDM-induced airway inflammation and asthma development.

### ADAMTS7 Negatively Regulates DC Effector Function and Th2 Immune Response

**Fig. 3:**
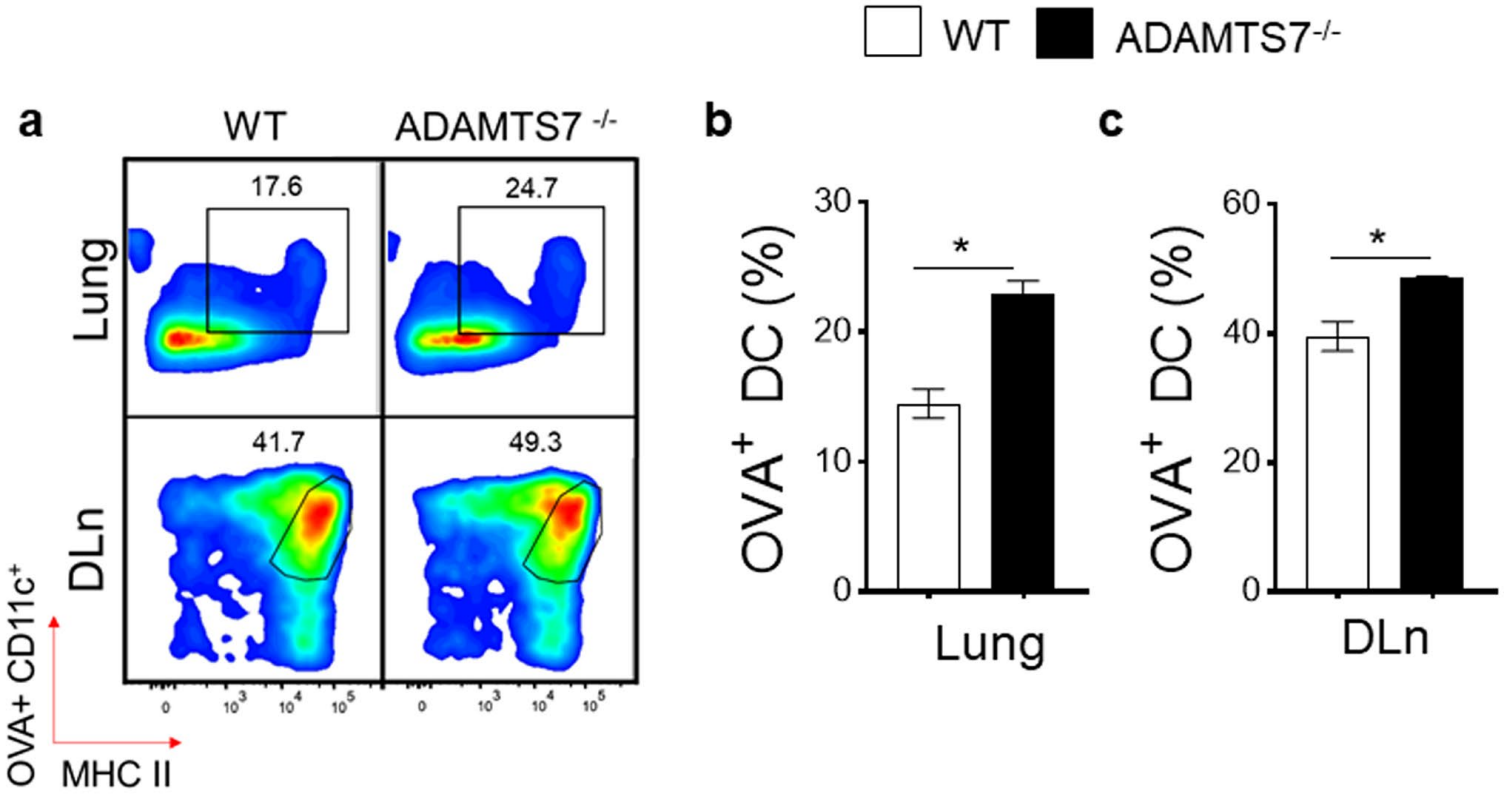
Antigen uptake by DC and transport to the lung DLn is regulated by ADAMTS7. **a** Shown are the representative FACS dot plots of HDM-challenged WT and ADAMTS7^−/−^ in the lung and DLn, and **b** the frequencies of OVA-AF647+CD11c+DCs (gated on SiglecF^−^ CD11c^+^ MHCII^hi^ population) from WT and ADAMTS7^−/−^ mice 72 h after the allergen administration. Results represent the mean ± S.E, pooled from two independent experiments (*n* = 3–4 mice per group, *P < 0.05 unpaired *t-*test)

Having observed an elevated phenotype of airway inflammation and Th2 adaptive immune response in ADAMTS7-deficient mice, we next assessed whether the antigen priming capabilities of ADAMTS7^−/−^ DC could interfere with the uptake and presentation of allergen as a possible mechanism. AF647-labeled OVA was administered intranasally to the WT and ADAMTS7^−/−^ lung, and the uptake/migration of OVA-loaded CD11c^+^ DC to DLn were enumerated by flow cytometry after 72 h (Fig. 3a). The frequencies of OVA bearing Alexa 647^+^ CD11c^+^ DC in the lung showed a significant increase in ADAMTS7^−/−^ mice compared with WT (Fig. 3b). In addition, significant differences were found between the genotypes in the frequency of CD11c^+^ OVA-AF647^+^ DC that migrated to the draining DLn after OVA antigen uptake by the lung (Fig. 3c). These experiments show that lung DC from ADAMTS7^−/−^ mice have an enhanced antigen uptake and migration capability compared to the WT mice.

**Fig. 4:**
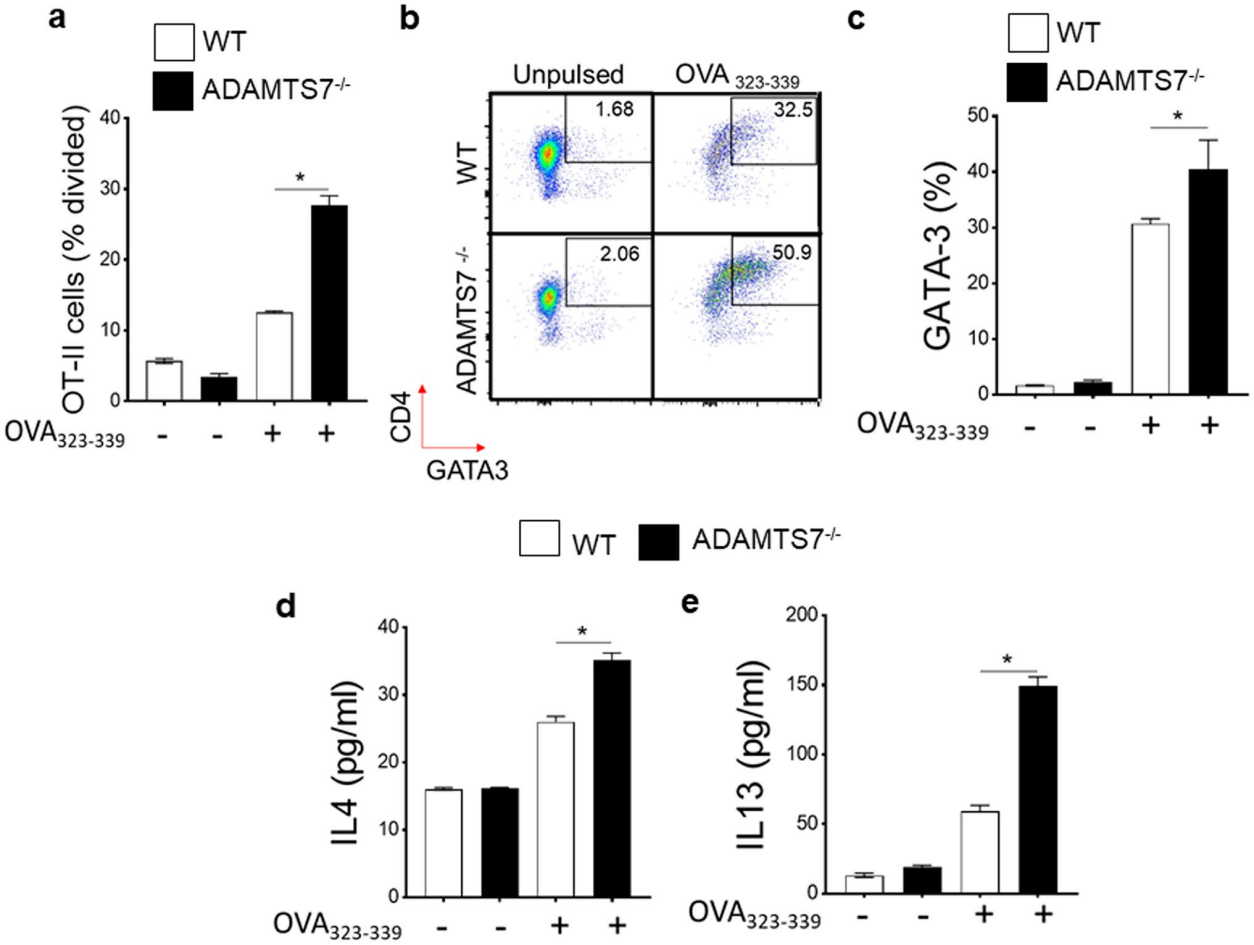
ADAMTS7 is a negative regulator of antigen priming and presentation function. **a** OVA-specific proliferation in OT-II cells are presented as a percentage divided. **b** Representative pseudocolor plots, **c** bar graphs of CD4^+^ GATA3^+^ OT-II cells, and Th2 cytokines **d** IL4 and** e** IL13 in BMDC-OTII coculture supernatant from WT and ADAMTS7^−/−^ with and without OVA_323-339_ peptide. (*n* = 6 per group and represent one of the three repeats). Data represent the mean ± SE, *P < 0.01, unpaired *t*-test

Coculture of CD4^+^ T-cells from OT-II transgenic mice that recognize the OVA_323-339_ peptide [20] with bone marrow-derived dendritic cells (BMDC) isolated from ADAMTS7^−/−^ mice displayed a marked increase in T-cell proliferation compared with BMDC isolated from WT mice (Fig. 4a). Moreover, GATA3 (a Th2-specific transcription factor) [23] expressions were markedly increased in OT-II cells with ADAMTS7^−/−^ BMDC compared with WT BMDC after OVA_323-339_ peptide stimulation (Fig. 4b, c). Similarly, BMDC from ADAMTS7^−/−^ mice showed augmented ability to induce Th2 cytokines, IL4, and IL13 productions compared with WT BMDC following OVA_323-339_ peptide stimulation when cocultured with splenic T-cells obtained from OT-II mice (Fig. 4d, e). These in vivo and ex vivo results collectively show that OVA-peptide-pulsed CD11c^+^ BMDCs from ADAMTS7^−/−^ mice have an elevated allergen uptake/migration and antigen-presenting capability to CD4+T-cells, thereby accelerating Th2 effector functions. Thus, increased uptake/migration of HDM antigen and enhanced antigen presentation and induction of Th2 immunity might contribute to the augmented eosinophilic airway inflammation phenotype in HDM-challenged ADAMTS7-deficient mice.

## Discussion

Lung DC plays pivotal roles in the initiation and perpetuation of Th2 cell differentiation and production of Th2 cytokine that mediate allergic airway inflammation and Th2 immune response [24–29]. We have previously shown that the regulation of T-cell-mediated lung adaptive immune responses are driven by antigen uptake/migration to the draining DLn and antigen priming capability of DC [7, 8, 30]. However, limited data exist regarding secretory proteoglycans ADAMTS7 in mediating the lung adaptive immune responses. We used an HDM-induced experimental murine model of allergic asthma to investigate whether ADAMTS7 expression in the lung modulates the subsequent induction and development of Th2 immune response to inhaled aeroallergen HDM. First, to characterize the role of ADAMTS7 in HDM-induced airway inflammation, we employed ADAMTS7-deficient transgenic mice and compared them with the WT control mice. ADAMTS7^−/−^ mice manifested elevated eosinophilic airway inflammation, mucous cell metaplasia, and Th2 immunity to HDM sensitization and challenge. Second, HDM-restimulation of DLn cells from ADAMTS7^−/−^ mice display a phenotype of augmented production of Th2 cytokines IL5, and IL13, which are critical regulators of eosinophilic airway inflammation and mucous metaplasia.

Structurally the disintegrin domain, seven thrombospondin repeats, cysteine-rich, and spacer domains following the catalytic domain regulate the substrate specificity and localization of ADAMTS7 [13, 31]. ADAMTS7 dosage and catalytic activity contribute to the atherosclerosis phenotype and are associated with coronary artery disease (CAD) risk [9, 14]. It has been posited that inhibiting the function of the ADAMTS7 coding variant (rs3825807; Ser214Pro) associated with CAD could mimic the cardioprotective effect. Moreover, elevated ADAMTS7 levels were found in rheumatoid arthritis patients [11]. However, the role of ADAMTS7 in lung inflammation has not been defined. By gating lung DC (SiglecF^−^ CD11c^+^ MHCII^hi^) populations, we show that ADAMTS7^−/−^ mice have enhanced antigen uptake/migration capabilities compared to WT control mice. The uptake of HDM antigen in the lung is predominantly mediated by the CD11c^+^ DC, whereas other CD11c^+^ antigen-presenting myeloid cells (e.g., alveolar macrophages) can also mediate HDM uptake at high doses [27, 32]. Notably, activation and migration of DC are negatively regulated in an autocrine fashion under inflammatory conditions by endogenous thrombospondin 1, which down-regulates proinflammatory cytokine production to terminate the antigenic response [33]. Following antigen uptake in the lung, DC are transported to draining DLn and induce expansion of naïve T-cells to mount a Th2 immune response [29, 34]. Moreover, the ex vivo coculture experiments demonstrate that ADAMTS7^−/−^ DC has increased antigen priming and presentation capability compared to the WT DC. Collectively, the results are consistent with the conclusion that ADAMTS7 plays essential lung functions as a negative regulator of DC, thereby attenuating HDM-induced allergic sensitization and eosinophilic airway inflammation.

The present study demonstrates, for what we believe is the first time, a causal role of ADAMTS7 in modulating DC function in the pathogenesis of Th2 immune responses to HDM in experimental allergen-induced asthma. We propose that the mechanism by which ADAMTS7 negatively regulates DC function involves attenuated antigen uptake and presentation capabilities, which reduces allergic sensitization and Th2 immune responses in the lung. Therefore, these findings support the role of the *ADAMTS7* gene as a negative regulator for DC-mediated allergic airway inflammation in asthma and provide a potentially novel treatment approach.

## Data Availability

All manuscript data can be made available by the corresponding author.
